# Bile components and lecithin supplemented to plant based diets do not diminish diet related intestinal inflammation in Atlantic salmon

**DOI:** 10.1186/s12917-016-0819-0

**Published:** 2016-09-07

**Authors:** Trond M. Kortner, Michael H. Penn, Ingemar Bjӧrkhem, Kjell Måsøval, Åshild Krogdahl

**Affiliations:** 1 Department of Basic Sciences and Aquatic Medicine, Faculty of Veterinary Medicine and Biosciences, Norwegian University of Life Sciences, Oslo, Norway; 2 Department of Laboratory Medicine, Division for Clinical Chemistry, Karolinska University Hospital, Huddinge, Sweden; 3 Biomar AS, Nordre gate 11, 7011 Trondheim, Norway; 4 Present Address: US Fish & Wildlife Service, Lamar, PA 16848 USA

**Keywords:** Gut health, Intestinal inflammation, Fish feed, Plant ingredients, Cholesterol, Bile

## Abstract

**Background:**

The present study was undertaken to gain knowledge on the role of bile components and lecithin on development of aberrations in digestive functions which seemingly have increased in Atlantic salmon in parallel with the increased use of plant ingredients in fish feed. Post smolt Atlantic salmon were fed for 77 days one of three basal diets: a high fish meal diet (HFM), a low fishmeal diet (LFM), or a diet with high protein soybean meal (HPS). Five additional diets were made from the LFM diet by supplementing with: purified taurocholate (1.8 %), bovine bile salt (1.8 %), taurine (0.4 %), lecithin (1.5 %), or a mix of supplements (suppl mix) containing taurocholate (1.8 %), cholesterol (1.5 %) and lecithin (0.4 %). Two additional diets were made from the HPS diet by supplementing with: bovine bile salt (1.8 %) or the suppl mix. Body and intestinal weights were recorded, and blood, bile, intestinal tissues and digesta were sampled for evaluation of growth, nutrient metabolism and intestinal structure and function.

**Results:**

In comparison with fish fed the HFM diet fish fed the LFM and HPS diets grew less and showed reduced plasma bile salt and cholesterol levels. Histological examination of the distal intestine showed signs of enteritis in both LFM and HPS diet groups, though more pronounced in the HPS diet group. The HPS diet reduced digesta dry matter and capacity of leucine amino peptidase in the distal intestine. None of the dietary supplements improved endpoints regarding fish performance, gut function or inflammation in the distal intestine. Some endpoints rather indicated negative effects.

**Conclusions:**

Dietary supplementation with bile components or lecithin in general did not improve endpoints regarding performance or gut health in Atlantic salmon, in clear contrast to what has been previously reported for rainbow trout. Follow-up studies are needed to clarify if lower levels of bile salts and cholesterol may give different and beneficial effects, or if other supplements, and other combinations of supplements might prevent or ameliorate inflammation in the distal intestine.

## Background

Levels of plant protein ingredients in feeds for salmonids and several other cultivated species have gradually increased over the last two decades, replacing fish meal. Substitution of marine with plant protein in feed for carnivorous fish, especially when using less refined plant protein sources such as full-fat or extracted soybean meal (SBM), will in many cases result in reduced body pools of cholesterol and bile acids [1–6]. In severe cases, the reduced levels of cholesterol and bile acids may be observed in concert with reduced fish growth and gastro-intestinal problems such as intestinal inflammation and steatosis [2, 7]. These conditions may be related to, or influenced by reduced levels of cholesterol and bile components, or disturbances in lipid digestion and metabolism. The apparent drain of bile acids in Atlantic salmon suffering from SBM-induced enteritis (SBMIE) [2, 3, 8, 9] is likely caused by a combination of the reduced dietary cholesterol load and specific action of soy antinutrients such as saponins, which may impair cholesterol and bile acid uptake from the intestinal lumen [10–13].

Recent reports suggest that bile salts, in addition to their key role in lipid digestion, have important anti-inflammatory effects in the gut, and can preserve the intestinal barrier in inflammatory bowel disease (IBD) models [14–17]. Similarly, studies with rainbow trout indicate that dietary inclusion of bile salts or soybean lecithin may prevent distal intestinal inflammation induced by SBM or plant antinutrients [5, 18, 19]. In Atlantic salmon, the SBMIE has been established as an excellent and reproducible intestinal inflammation model, and the Atlantic salmon is more susceptible to SBMIE than the rainbow trout. However, it has not yet been investigated if supplementation with bile components or lecithin can prevent or reduce diet related intestinal inflammation in Atlantic salmon.

The work presented here is part of a larger experimental series with the main objective to develop knowledge needed to produce sustainable, healthy and cost efficient fish feeds with low fish meal inclusion, based on plant and other alternative nutrient sources, and to identify indicators of feed related health effects. Specifically, the present study aimed at increasing knowledge on relationships between plant induced intestinal inflammation and deficiencies of bile components in Atlantic salmon. Based on the above mentioned studies in rainbow trout, we hypothesized that dietary supplementation with bile components or lecithin to plant protein based diets would improve performance and gut health in Atlantic salmon. For that purpose, a 77 day feeding trial was conducted. At termination of the feeding trial, the weights of body and intestines were recorded, and blood, bile, intestinal tissues and digesta were sampled for evaluation of effects on growth, nutrient metabolism and intestinal structure (histomorphological changes) and function (digestive enzyme activities and faecal dry matter).

## Methods

### Experimental animals, diet and sampling

Atlantic salmon (*Salmo salar* L.) post smolts of the Sunndalsøra breed with mean weight of 362 ± 95 g (mean ± SD) were weighed, pit tagged and randomly allocated into 20 cylindrical fiberglass tanks (200 L, 35 fish pr tank) with flow-through seawater (6–7 L min^−1^). Two replicate tanks per diet were used. Water temperature varied between 7 and 14 °C. Oxygen content and salinity of the outlet water were monitored to ensure saturation above 85 % and stability, respectively. A 24 h lighting regime was employed during the experimental period. The fish were weighed individually when allocating the fish to the experimental units to assure similar biomass in all tanks.

Ten experimental diets were formulated (Table 1). A fish meal based diet (high fish meal; HFM) was used as a control. A low fish meal (LFM) combination of soy protein concentrate (SPC) and pea protein concentrate, or high protein soya (HPS) provided the bulk of dietary protein in the two other diets. Conjugated bile salts, taurine, lecithin and cholesterol were added to these diets singly or in combination as described in Table 1. Supplementation levels were based on levels used in rainbow trout as previously reported [5, 18, 19]. Feed intake was not recorded. Diets were formulated to contain 41 % crude protein and 30 % lipid (DM basis). They were supplemented with a standard vitamin and micro-mineral premix and limiting essential amino acids (lysine, methionine) as necessary to provide required amounts as suggested by NRC guidelines [20]. Diets also contained 100 mg kg-1 yttrium oxide as an inert marker for calculation of nutrient apparent digestibilities. Chemical analysis of the diets is shown in Table 1. Feed was produced by extrusion at the BioMar AS production facility in Brande, Denmark. Diets were extruded with a feed pellet size of 6 mm.

**Table 1:** Diet formulation and chemical analysis

	HFM	LFM	LFM +	LFM +	LFM +	LFM +	LFM +	HPS	HPS +	HPS +
			Tauro-cholate	Bovine bile salt	Taurine	Lecithin	Suppl Mix		Bovine bile salt	Suppl Mix
*Ingredient (g 100 g* ^*−1*^ *)*										
SA SP Sara Rousing^a^	15.00	5.00	5.00	5.00	5.00	5.00	5.00	7.50	7.50	7.50
Nordic LT 94 fishmeal^b^	15.00	5.00	5.00	5.00	5.00	5.00	5.00	7.50	7.50	7.50
Soya HP 48^c^								20.00	20.00	20.00
Soya 60 % (SPC)^d^	10.00	19.03	19.03	19.03	19.03	19.03	19.03	3.52	3.52	3.52
Corn Gluten^e^	5.34	15.00	15.00	15.00	15.00	15.00	15.00	10.19	10.19	10.19
Pea Protein 50^f^	6.00	13.00	13.00	13.00	13.00	13.00	13.00	0.40	0.40	0.40
Dehulled Beans^g^	14.58	14.00	14.00	14.00	14.00	14.00	14.00	14.00	14.00	14.00
Sunflower expeller^h^	10.00									
Wheat Gluten^i^		1.97	1.97	1.97	1.97	1.97	1.97	10.00	10.00	10.00
Fishoil (Standard)^j^	7.45	7.67	7.67	7.67	7.67	7.67	7.67	7.82	7.82	7.82
Rapeseed oil^k^	17.09	17.60	17.60	17.60	17.60	17.60	17.60	17.94	17.94	17.94
Sodium taurocholate^l^			1.80							
Bovine bile salt^l^				1.80			1.80		1.80	1.80
Cholesterol^l^							1.50			1.50
Taurine^l^					0.40					
Lecithin^l^						1.50	0.40			0.40
MCP	0.70	1.82	1.82	1.82	1.82	1.82	1.82	1.56	1.56	1.56
Lysine	0.20	0.81	0.81	0.81	0.81	0.81	0.81	0.98	0.98	0.98
Methionine	0.11	0.32	0.32	0.32	0.32	0.32	0.32	0.25	0.25	0.25
Threonine		0.11	0.11	0.11	0.11	0.11	0.11	0.14	0.14	0.14
Barox (antioxidant)	0.03	0.03	0.03	0.03	0.03	0.03	0.03	0.03	0.03	0.03
Vitamin-Mineral mix^m^	0.30	0.30	0.30	0.30	0.30	0.30	0.30	0.30	0.30	0.30
Yttrium^n^	0.05	0.05	0.05	0.05	0.05	0.05	0.05	0.05	0.05	0.05
Lucantin Pink CWD 10 %	0.04	0.04	0.04	0.04	0.04	0.04	0.04	0.04	0.04	0.04
*Chemical analysis* ^o^										
Dry matter (%)	95.0	95.0	95.0	95.0	95.0	95.0	95.0	95.0	95.0	95.0
Ash (%)	7.0	5.0	5.0	5.0	5.0	5.0	5.0	5.2	5.2	5.2
Fat (%)	29.8	29.2	29.2	29.2	29.2	29.2	29.2	29.6	29.6	29.6
Protein (%)	40.8	40.4	40.4	40.4	40.4	40.4	40.4	41.4	41.4	41.4
D Protein (%)	36.0	36.0	36.0	36.0	36.0	36.0	36.0	36.0	36.0	36.0
DE (MJ/kg)	20.3	20.3	20.3	20.3	20.3	20.3	20.3	20.3	20.3	20.3
GE (MJ/kg)	24.4	24.4	24.4	24.4	24.4	24.4	24.4	24.6	24.6	24.6
Cholesterol (g/kg)	2.1	0.8	0.7	0.6	0.7	0.7	19.1	1.0	1.0	17.8
Bile salts (g/kg)	0.3	0.2	16.3	n/a	n/a	n/a	11.0	n/a	10.58	n/a

^a^Superprime, supplied by Köster Marine Proteins GmbH, Hamburg, Germany

^b^Supplied by Norsildmel AS, Bergen, Norway

^c^Supplied by Scan Mills, Germany

^d^Supplied by Selecta S/A, Av. Jamel Ceilio, 2496 – 12th region, Goiania, Brazil

^e^Supplied by Cargil Nordic, SAS van Gent, Holland

^f^Supplied by DLG Food Grain, Roslev, Denmark

^j^Supplied by HC Handelscenter, Skibby, Denmark

^h^Supplied by DLA agro, Denmark

^i^Supplied by Roquette, Beinheim, France

^j^Supplied by FF Skagen, Skagen, Denmark

^k^Supplied by Emmelev, Otterup, Denmark

^l^Supplied by Sigma-Aldrich, Broendby, Denmark

^m^Supplied to meet requirements. Composition is intellectual property of BioMar AS

^n^Inert marker for the evaluation of nutrient digestibility

^o^Complete chemical analysis was conducted only for the basal diets (HFM, LFM, HPS). Cholesterol content was analyzed in all diets, bile salt content was analyzed in five diets as shown in table

The feeding trial ran for 77 days. Tank sampling order and fish sampling were conducted randomly. Fifteen fish were sampled from each tank and euthanized by anaesthetization with tricaine methane-sulfonate (MS-222) followed by a sharp blow to the head. From ten fish per tank, blood was sampled by venipuncture of the caudal vein. Blood was collected in Vacutainers containing lithium heparin and stored on ice until centrifugation. Plasma was separated and immediately frozen in liquid nitrogen and stored at −80 °C until analysis. After blood withdrawal, fish were dissected to remove the viscera. Intestinal contents (digesta) were collected from the pyloric, mid and distal intestines. The contents from the pyloric intestines were divided into two equal portions labelled as PI1 and PI2 where PI1 constituted the most proximal located portion. A similar separation was performed for the contents of the distal intestines and labelled as DI1 and DI2. Intestinal contents were frozen in liquid nitrogen and stored at −80 °C until analysis. For analysis of leucine amino peptidase enzymatic activity, the entire pyloric caeca and distal intestine tissues were immediately frozen in liquid nitrogen in pre-weighed tubes and stored at −80 °C before further processing. From five additional fish per tank, distal intestine tissues were fixed in 10 % neutral buffered formalin (4 % formaldehyde) for 24 h and subsequently transferred to 70 % EtOH for storage until processing for histological examination. The remaining fish in each tank were stripped for faeces and continued on feeds for an additional week at which time they were stripped again. Faecal samples were pooled and frozen until analysis.

### Chemical analyses

Diet and faecal samples were analyzed for dry matter (after heating at l05°C for 16–18 h), ash (combusted at 550 °C to constant weight), nitrogen (crude protein) (by the semi-micro-Kjeldahl method, Kjeltec-Auto System, Tecator, Höganäs, Sweden), fat (diethylether extraction in a Fosstec analyzer (Tecator) after HCl-hydrolysis), starch (measured as glucose after hydrolysis by alpha-amylase (Novo Nordisk A/S, Bagsvaerd, Denmark) and amylo-glucosidase (Bohringer Mannheim GmbH, Mannheim, Germany), followed by glucose determination by the ‘Glut-DH method’ (Merck, Darmstadt, Germany)), gross energy (using the Parr 1271 Bomb calorimeter, Parr, Moline, IL, USA), and yttrium (by inductivity coupled plasma (ICP) mass-spectroscopy as described by Refstie et al. [21]).

### Plasma variables and bile salt levels

All diets were analyzed for cholesterol by isotope dilution mass spectrometry as described by Schaffer et al. [22]. Plasma was analyzed for cholesterol following standard procedures at the Central Laboratory of the Norwegian University of Life Sciences, Faculty of Veterinary Medicine and Biosciences, Oslo. Total intestinal bile salt levels were measured in plasma and pooled freeze dried gastrointestinal contents from PI1, PI2, MI, DI1, and DI2. Bile salt concentration was determined using the enzyme cycling amplification/Thio – NAD method (Inverness Medical, Cheshire, UK) in the ADVIA®1650 Chemistry System (Siemens Healthcare Diagnostics Inc.) at the Central Laboratory. In diet samples and bile taken directly from the gall bladder, glycine and taurine conjugated bile acids were analyzed by HPLC-MS-MS by a modification of the method described by Tagliacocci et al. [23] using deuterium labeled glycine derivatives of bile acids as internal standards. Total bile acids in plasma and in intestinal contents were also analyzed by isotope dilution and combined GC-MS after addition of deuterated cholic acid, chenodeozycholic acid and deoxycholic acid as internal standards followed by deconjugation as described by Björkhem and Falk [24]. The two methods have been shown to give almost identical results. Plasma oxysterols were analyzed by isotope dilution and combined GC-MS after hydrolysis as described by Dzeletovic et al. [25]. Sitosterol and campesterol were assayed by isotope dilution and combined GC-MS after hydrolysis as described by Acimovic et al. [26]. Lathosterol was analyzed by isotope dilution mass spectrometry as described by Lund et al. [27]. 7α-hydroxy-4-cholesten-3-one (C4) was analyzed by isotope dilution and use of combined HPLC-MS as described by Lövgren-Sandblom et al. [28]. Lipoprotein profiles in plasma were conducted employing size exclusion chromatography and measurements of cholesterol on-line using microliter sample volumes as described by Parini et al. [29].

### Histology

Formalin fixed DI tissue samples were processed using standard histological techniques and stained with haematoxylin and eosin (H&E). Examination was conducted blinded and in randomized order. The degree of histomorphological change (i.e., deviation from normal) was assessed and assigned to one of four categories: normal, slight, moderate or marked. The following histological characteristics were evaluated: length and fusion of mucosal folds, cellular infiltration and width of the lamina propria and submucosa, enterocyte vacuolization, nucleus position within the enterocytes and the relative number of goblet cells [30, 31].

### Calculations

Crude protein (CP) was calculated as N x 6.25. Thermal-unit growth coefficient (TGC) was calculated as: TGC = 1000*(FBW^1/3^ – IBW^1/3^) x (ΣD°)^−1^, where IBW and FBW are the initial and final body weights (tank means) and ΣD° is the thermal sum (feeding days × average temperature in °C). The specific growth rate (SGR) was calculated using the tank means for initial body weight (IBW) and final body weight (FBW) as follows: SGR = [(ln FBW – ln IBW) /number of days] × 100. Organosomatic indices were calculated as percentages of the weight of the organ in relation to body weight.

### Statistical analyses

Data was analyzed using one-way ANOVA followed by Duncan’s test for post hoc comparison. Tank means were used as the statistical unit. Histology data from individual fish were analyzed using Chi-square test. The level of significance was set to *p* < 0.05 for all analyses.

## Results

### Diet content of cholesterol and bile salts

As expected, among the diets used in this experiment cholesterol level was higher in the HFM diet than in the LFM and HPS basal diets. Supplementation with cholesterol (suppl mix) increased diet cholesterol concentration (Table 1). Bile salt concentrations were determined in five diets: HFM, LFM, LFM + taurocholate, LFM + suppl mix, and HPS + bovine bile salt diets (Table 1). Unsupplemented diets contained very little bile salts, and as expected the LFM had lower levels than the HFM diet. Supplementation with bile salts, either taurocholate or bovine bile salt, markedly increased dietary bile salt level. The taurocholate supplement was more than 98 % pure, a result that was confirmed by direct analysis (data not shown). The bovine bile salt used as supplement contained a range of bile acids and bile salts. Free bile acids, tauroconjugates and glycoconjucates comprised about 45, 25 and 30 % respectively of this bovine bile preparation.

### Fish growth, organ indices and nutrient digestibilities

Final fish weights, thermal growth coefficients (TGC) and specific growth rates (SGR) are presented in Table 2. TGC and SGR values were significantly lower in fish in the groups fed the LFM and HPS diets compared to those receiving the HFM diet. None of the supplements improved TGC or SGR significantly. The lowest TGC and SGR values were found in the fish fed the bovine bile salt supplemented diets (LFM + bovine bile salt, LFM + suppl mix, HPS + bovine bile salt, and HPS + suppl mix). When supplemented to the LFM diet, the reductions in TGC and SGR values were significant.

**Table 2:** Growth and relative organ weights of Atlantic salmon during the feeding period

	HFM	LFM	LFM +	LFM +	LFM +	LFM +	LFM +	HPS	HPS +	HPS +	
			Tauro-cholate	Bovine bile salt	Taurine	Lecithin	Suppl Mix		Bovine bile salt	Suppl Mix	Pooled SEM
BW (g)	789^a^	709 ^bcd^	678^cd^	656 ^cd^	704 ^bcd^	758 ^ab^	638^d^	681 ^cd^	669 ^cd^	667^cd^	17
TGC	3.04^a^	2.54^bcd^	2.36^cde^	2.09^e^	2.52 ^bcd^	2.75^ab^	2.08^e^	2.37^bcde^	2.24^cde^	2.16^de^	0.09
SGR	1.02^a^	0.87^bc^	0.81^bcd^	0.72^d^	0.86^bc^	0.92^ab^	0.72^d^	0.81^bcd^	0.77^cd^	0.74^d^	0.03
*Organosomatic indicies*											
PI	2.02^c^	2.52^a^	2.37^ab^	2.30^abc^	2.33 ^abc^	2.12^bc^	2.41^ab^	2.01^c^	2.09^bc^	2.09^bc^	0.08
MI	0.17^ef^	0.21^ab^	0.22^a^	0.21^abc^	0.19 ^cd^	0.17^f^	0.19^cd^	0.17^ef^	0.19 ^de^	0.19^def^	0.005
DI	0.48^ab^	0.49^a^	0.47^abcd^	0.43^de^	0.48 ^abc^	0.49^ab^	0.44^bcde^	0.39^e^	0.43 ^cde^	0.40^e^	0.01

Abbreviations: *BW* body weight, *TGC* thermal growth coefficient, *SGR* specific growth rate, *PI* pyloric intestine, *MI* mid intestine, *DI* distal intestine. Different letters denote diet groups that are significantly different

Organosomatic indices of the pyloric (PI), mid (MI) and distal (DI) intestines are shown in Table 2. For PI somatic indices significant different treatment effects were observed. The LFM fed fish had significantly higher PI somatic index compared to those fed the HFM and HPS diets. Among the LFM groups of fish, lecithin supplementation led to lower relative PI weights. No differences due to supplementation were observed among the HPS groups. Similar results were observed in the MI, increased weights in the LFM groups compared to HFM and HPS groups, except for the lecithin supplemented groups, which showed similar values as HFM and HPS groups. The opposite situation was observed in the distal intestine. LFM fed fish had similar DI somatic index as those fed HFM, but the HPS groups had a significantly lower DI relative weight. Among the LFM groups, fish fed diets supplemented with bovine bile salt (LFM + bovine bile salt and LFM + suppl mix) had lower DI somatic indices compared to the non-supplemented fish (LFM). The supplementations to the HPS diet did not significantly affect relative weight of DI.

Table 3 shows the results of the digestibility analyses. Only small differences were observed for the protein and lipid digestibilities. Interestingly enough, the LFM diets showed significantly higher protein digestibility than the HFM diet. No significant differences were observed between the LFM and HPS diets. Likewise, no significant differences in lipid digestibility were observed between these diets.

**Table 3:** Apparent digestibility of crude protein and lipid

	HFM	LFM	LFM +	LFM +	LFM +	LFM +	LFM +	HPS	HPS +	HPS +	
			Tauro-cholate	Bovine bile salt	Taurine	Lecithin	Suppl Mix		Bovine bile salt	Suppl Mix	Pooled SEM
Protein	88.3^c^	90.0^ab^	89.4^abc^	89.0^bc^	90.1^ab^	90.7^a^	89.5^abc^	90.2^a^	90.0^ab^	89.3^bc^	0.25
Lipid	96.5^a^	95.7^abc^	95.0^bc^	95.7^abc^	95.2^abc^	96.2^ab^	94.5^c^	95.1^bc^	96.2^ab^	94.6^c^	0.25

Different letters denote diet groups that are significantly different

### Morphology of the distal intestine

**Fig. 1:**
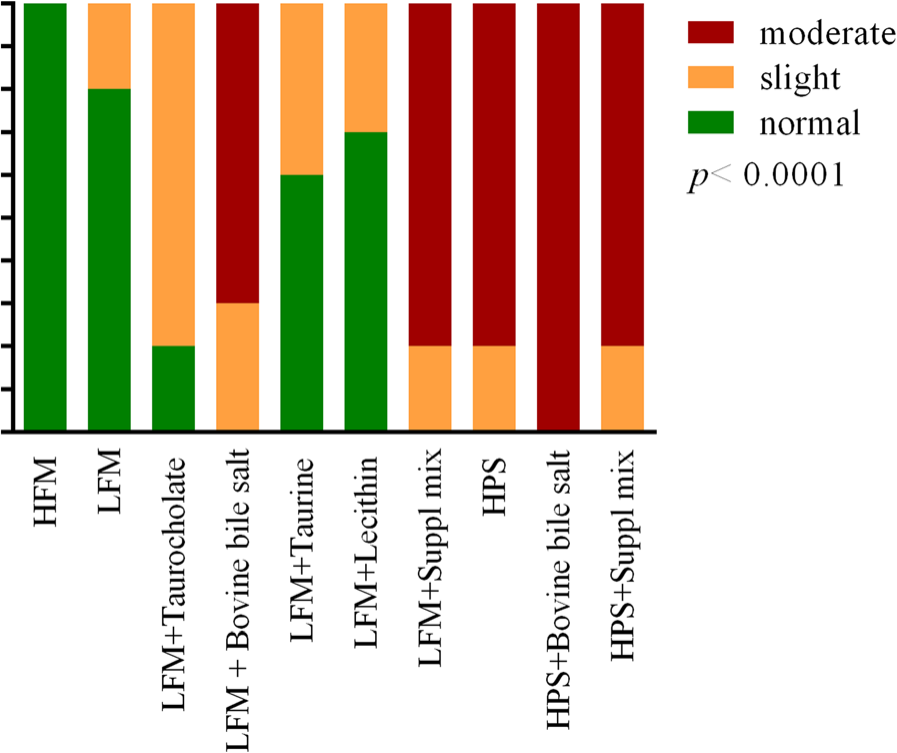
Number of samples in each diet group classified by severity of inflammatory changes in the distal intestine. The *P* value for the Chi-square test is given

**Fig. 2:**
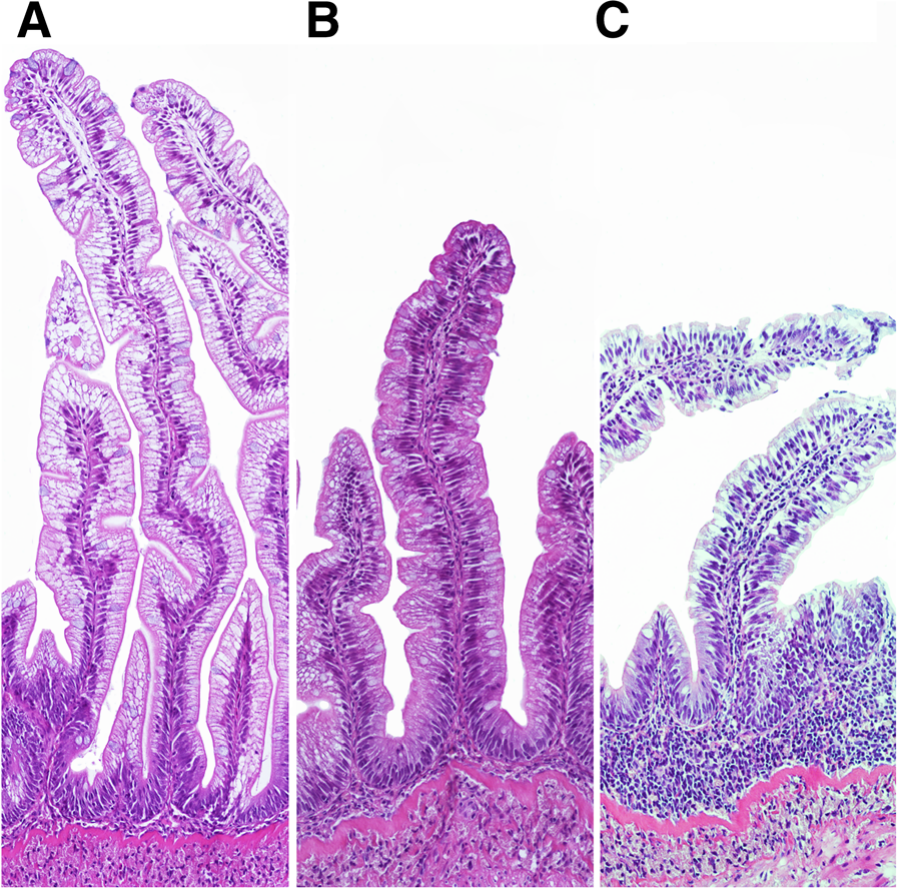
Distal intestinal histolomorphology showing representative appearance of sections that were graded as (**a**) normal, (**b**) mild, or (**c**) moderate changes characteristic of soybean meal-induced distal intestinal enteritis

**Fig. 3:**
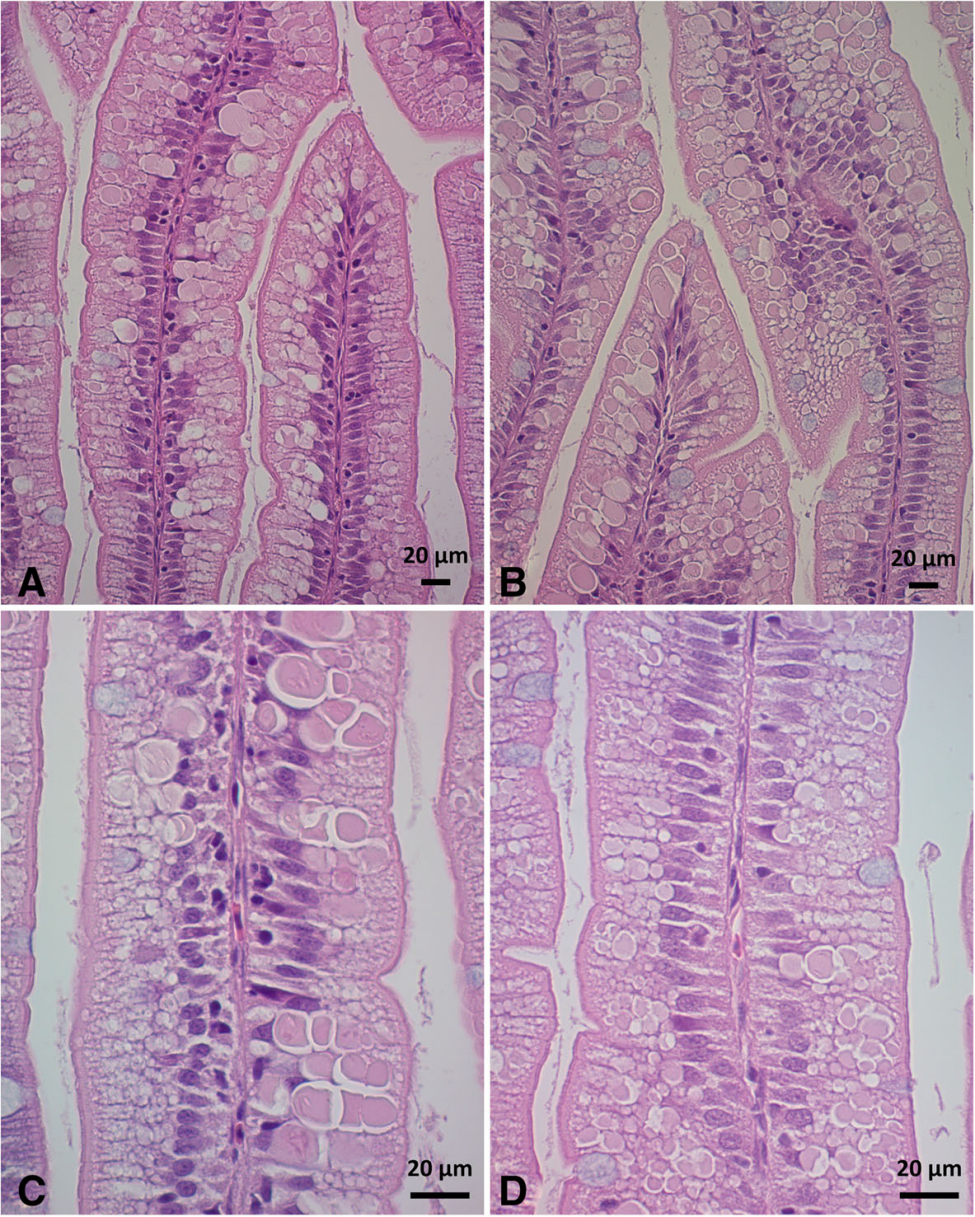
Eosinophilic inclusions within distal intestine enterocytes in (**a**) LFM, (**b**) LFM + taurocholate, (**c**) LFM, and (**d**) LFM + taurocholate fed fish

Slight to moderate inflammatory changes were observed in several samples. See Fig. 1 for numbers of samples from each treatment classified by severity of changes and Fig. 2 for representative histological images. All fish fed the HFM diet appeared normal. Eight out of ten fish fed the LFM diet appeared normal, whereas 2 fish showed slight changes. Supplementing the LFM diet with taurocholate increased the number of samples with slight changes, whereas supplementing the LFM diet with bovine bile salt or the suppl mix clearly increased the number of samples with moderate changes. Other LFM diets did not significantly affect the number of samples with inflammatory changes, or the severity of changes. Varying degrees of accumulation of eosinophilic material within enterocytes (Fig. 3) were frequently observed in fish fed LFM diets. Fish fed the LFM + taurocholate diet had the highest frequency of eosinophilic inclusions, in 8 out of 10 samples.

Inflammatory responses varied between fish fed HPS-containing diets. When present, changes were typical of soy enteropathy, including decreased enterocyte vacuolization, apical displacement of enterocyte nuclei, leukocyte infiltration of the epithelia and submucosa, and hyperplastic connective tissue in the lamina propria and submucosa. Supplementation of the HPS diet with either the bovine bile salt or the suppl mix did not significantly affect the number of fish showing inflammatory changes, or the severity of changes.

### Blood plasma biochemistry

Blood plasma variables are presented in Table 4. Total plasma cholesterol levels were higher in fish fed cholesterol supplemented diets (suppl mix). No significant differences were observed between fish fed the other diets. Most of the plasma cholesterol was present in the HDL lipoprotein fraction, except in the fishes fed with the cholesterol supplemented diets (suppl mix). In these, most of the cholesterol was present in the LDL fraction and much less in the HDL fraction.

**Table 4:** Mean values (*n* = ten fish pr diet group) for blood plasma variables

	HFM	LFM	LFM+ Tauro cholate	LFM+ Bovine bile salt	LFM+ Taurine	LFM+ Lecithin	LFM+ Suppl mix	HPS	HPS+ Bovine bile salt	HPS+ Suppl mix	pooled SEM
*Total CH (mmol/l)*	10.7^a^	8.3^a^	8.5^a^	8.4^a^	8.4^a^	9.5^a^	24.6^b^	8.6^a^	8.9^a^	24.6^b^	1.1
VLDL-CH^a^	0.1	0.1	0.2	0.2	0.2	0.1	0.5	0.2	0.1	0.5	
LDL-CH^a^	1.6	1.3	0.9	1.0	0.9	0.9	19.9	0.9	1.0	23.5	
HDL-CH^a^	10.5	7.5	6.6	7.9	6.4	8.0	7.3	7.5	5.8	9.1	
Bile salts (μmol/l)	31^abc^	20^bc^	23^abc^	48^ab^	22^bc^	15^bc^	55^a^	6^c^	41^ab^	23^abc^	7
Lathosterol (μg/ml)	6.6^b^	3.8^cd^	3.2^de^	3.5^d^	4.7^c^	4.6^c^	2.4^ef^	5.8^b^	3.6^d^	2.5^ef^	0.4
C4 (ng/ml)	13^a^	10^a^	10^a^	10^a^	13^a^	11^a^	200^b^	12^a^	9^a^	180^b^	8
Sitosterol(μg/ml)	67^ab^	74^a^	47^cd^	52^bcd^	44^d^	64^ab^	6^e^	64^ab^	40^d^	6^e^	4
Campesterol(μg/ml)	229^a^	188^ab^	151^b^	160^b^	133^b^	220^a^	14^c^	239^a^	162^b^	18^c^	16
*Oxysterols (ng/ml)* ^a^											
7α-hydroxy-CH	124	130	111	116	172	116	1800	103	591	2110	
7β-hydroxy-CH	52	37	40	42	66	29	238	32	404	285	
7-keto-hydroxy-CH	130	101	122	125	201	59	730	89	1915	554	
24-hydroxy-CH	3.6	2.2	1.8	2.0	3.3	3.5	7.1	3.8	5.3	11.5	
25-hydroxy-CH	5	5	7	8	9	7	36	5	15	40	
27-hydroxy-CH	34	21	14	17	42	22	64	32	22	92	

^a^Lipoprotein and oxysterol profiles were measured in a pooled sample of *n* = ten animals pr diet group. CV for the different assays, i.e., the analytical variance, as estimated by analyzing a control sample over 10 consecutive days: VLDL-CH: 8.1 %, LDL-CH: 3.4 %, HDL-CH: 5.0 %, VLDL-TAG: 13.1 %, LDL-TAG: 10.5 %, HDL-TAG: 9.7 %. CVs for all oxysterol assays are <8 %, except for 25-hydroxy-CH (11 %) ^(25)^. Different letters denote diet groups that are significantly different

Plasma bile salt concentrations differed between treatments. The individual variation of plasma bile salt was, however, greater than expected, in particular for the groups fed bovine bile salt. The variation was largely reflective of dietary supplementation with bile acids. Although the One-Way ANOVA did not show significant differences between fish fed any of the basal diets, taking the results of all the LFM treatments without bile salt supplementation together it is clear that fish fed LFM diet had lower plasma total bile salt levels than fish fed the HFM diet. The HPS groups had the lowest plasma bile salt concentration. In fish fed both LFM and HPS, the bovine bile salt and suppl mix groups had higher levels of plasma bile acids compared to groups fed their respective basal diets.

Lathosterol is an intermediate in cholesterol synthesis and the circulating level of this steroid reflects cholesterol synthesis in the liver. As expected, the cholesterol-containing diets (suppl mix) depressed circulating lathosterol. Lower lathosterol levels were also observed in the LFM groups as compared to HFM and HPS groups. The cholesterol supplemented diets caused markedly increased plasma levels of 7α-Hydroxy-4-cholesten-3-one (C4), indicative of conversion of excess cholesterol to bile acids. No significant differences in C4 levels were observed between fish fed the other diets. Marked reduction in plasma levels of the plant sterols sitosterol and campesterol were observed for fish fed the cholesterol-containing diets (suppl mix). Supplementation with taurocholate, taurine and the bovine bile salt also reduced plasma plant sterol levels, but to a lesser extent. Plasma levels of oxysterols were markedly higher in the two cholesterol supplemented groups, whereas few differences were observed between fish fed the other diets.

### Gall bladder bile

Bile taken directly from the gall bladder was analyzed for individual bile salts. Total, conjugated and unconjugated bile salt concentrations are shown in Table 5. No significant differences in total concentrations were found between the LFM and HPS fed groups of fish compared to the HFM fed fish. Bovine bile salt supplementation increased or tended to increase total bile acid concentrations when added to both the LFM and HPS diets. The majority of bile acids in the gallbladder bile were conjugated; the only unconjugated bile acid found was cholic acid which was detected at low concentration. The taurine conjugated bile acids were the predominant form of bile acids found in the bile, with taurocholic acid being the predominant individual bile acid. Taurodeoxycholic acid was higher in the bile of fish groups fed diets supplemented with the bovine bile salt. The glycine conjugated bile acids were detected at very low concentrations except for the groups fed diets supplemented with bovine bile salt. The glycine conjugated bile acids were also largely responsible for differences observed in total bile acid concentrations since no statistically significant differences were observed in total taurine conjugated bile acids.

**Table 5:** Mean values (*n* = ten fish pr diet group) for gall bladder bile acid levels

	HFM	LFM	LFM+ Tauro cholate	LFM+ Bovine bile salt	LFM+ Taurine	LFM+ Lecithin	LFM+ Suppl mix	HPS	HPS+ Bovine bile salt	HPS+ Suppl mix	pooled SEM
Total bile acids	99^cd^	117^bcd^	112^bcd^	137^ab^	104^bcd^	95^d^	139^ab^	112^bcd^	161^a^	134^abc^	9
Total conjugated	99^cd^	117^bcd^	112^bcd^	136 ^ab^	104^bcd^	95^d^	138 ^ab^	112^bcd^	161^a^	133^abc^	9
Total unconjugated	<0.1^c^	<0.1^c^	<0.1^c^	0.6^b^	<0.1^c^	<0.1 ^c^	1.2^a^	<0.1^c^	0.3^bc^	1.0^a^	<0.1
Taurine conjugated	99^cd^	117^bcd^	112^bcd^	97^cd^	104^bcd^	95^d^	113^bcd^	112^bcd^	122^b^	107^bcd^	8
*T-CA*	92^ab^	110^a^	108^a^	66^b^	99^ab^	89^ab^	92^ab^	100^ab^	87^ab^	83^ab^	9
*T-CDCA*	7^bc^	6^c^	2^d^	6^c^	5^cd^	6^c^	9^bc^	13^a^	7^bc^	10^ac^	1
*T-DCA*	<0.1^c^	<0.1^c^	2^c^	25^a^	<0.1^c^	<0.1^c^	12^b^	<0.1^c^	28^a^	14^b^	1
Glycine conjugated	<0.1^c^	<0.1^c^	<0.1^c^	39^a^	<0.1^c^	<0.1^c^	25^b^	0.1^c^	39^a^	26^b^	1
*G-CA*	<0.1^c^	<0.1^c^	<0.1^c^	27^a^	<0.1^c^	<0.1^c^	19^b^	0.1^c^	30^a^	19^b^	1
*G-CDCA*	<0.1^d^	<0.1^d^	<0.1^d^	1.9^a^	<0.1^d^	<0.1^d^	0.8^c^	<0.1^d^	1.4^b^	0.9^c^	<0.1
*G-DCA*	<0.1^d^	<0.1^d^	<0.1^d^	9.8^a^	<0.1^d^	<0.1^d^	5.3^c^	<0.1^d^	8.1^ab^	6.1^bc^	0.3

Abbreviations: *T-* tauro-, *G-* glyco, *CA* colic acid, *CDCA* chenodeoxycholic acid, *DCA* deoxycholic acid. Different letters denote diet groups that are significantly different

### Brush border membrane leucine aminopeptidase activity

**Fig. 4:**
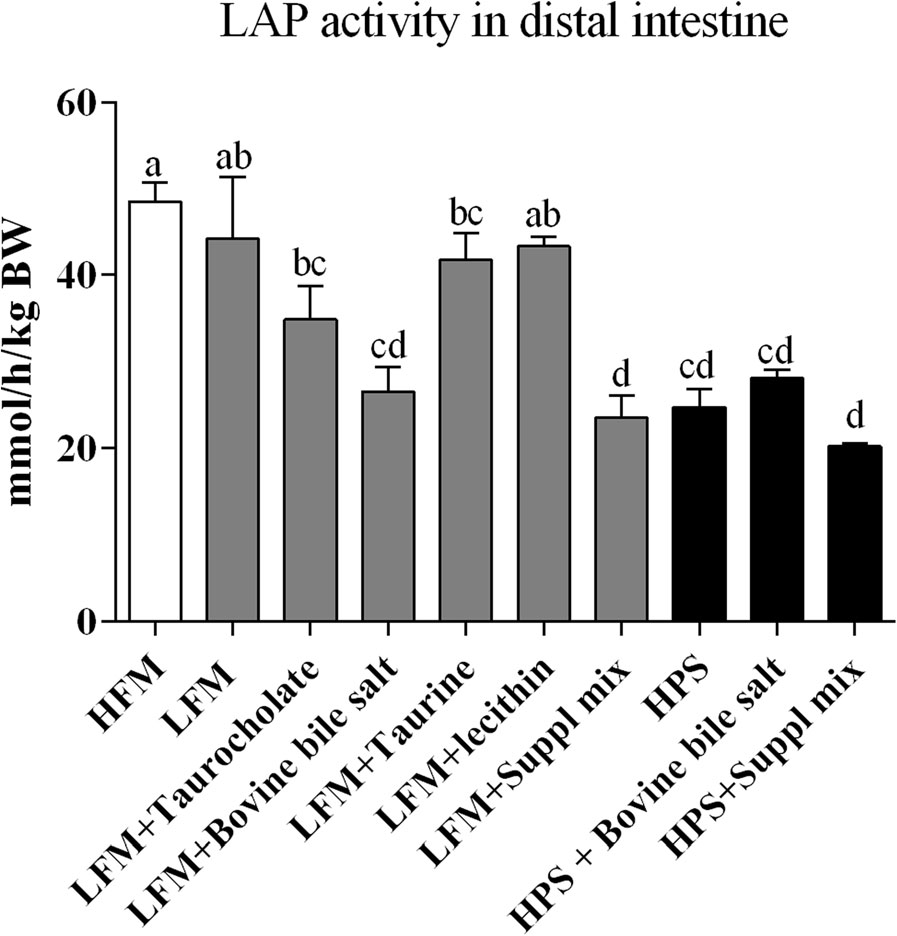
Leucine aminopeptidase (LAP) activity in the distal intestinal tissue, expressed as per kg body weight. Values are means with standard errors represented by vertical bars. Different letters denote diet groups that are significantly different

Leucine aminopeptidase activities were analyzed in pyloric and distal intestine tissue and expressed as total activity per kg fish weight. In the PI, no significant effects of basal diet formulation or any of the supplementations were found (data not shown). In the DI, the HPS groups showed lower enzyme activity compared to the HFM and LFM groups (Fig. 4). Groups fed diets with the suppl mix, i.e., both the LFM and HPS groups, showed the lowest activities. Fish fed the diets with bovine bile salt also showed lower enzyme activity compared to the LFM control. Supplementation with either bovine bile salt or suppl mix to the HPS formulation did not result in significant effects regarding this enzyme activity compared to the respective unsupplemented diets.

### Dry matter, trypsin activity and bile salt levels in intestinal content

Dry matter (DM) of digesta in the intestinal sections (PI1, PI2, MI, DI1, DI2) are presented in Table 6. No statistically significant differences were found in DM of digesta from the PI and MI. However, the trends observed in these regions became significant in the DI. The digesta DM of fish fed the LFM diet did not differ significantly from those fed the HFM diet, but the HPS groups showed significantly lower digesta DM in both the DI segments. For the LFM fed groups, bile salt supplementation tended to decrease DM. No differences due to any of the supplementation were observed among the HPS diets. Trypsin activities in intestinal regions are shown in Table 6. In the distal half of the DI, trypsin activities in fish fed the LFM diet were low and similar to fish fed the HFM diet. Fish fed the HPS diet showed significantly higher trypsin activity as compared to the other controls. Fish fed bile salt (taurocholate, bovine bile salt and suppl mix) supplemented LFM diets had higher trypsin activity compared to their respective control and similar to the HPS groups. Levels were high in all HPS groups, and no effect of supplementation was observed. No statistically significant differences in digesta bile salt concentrations were observed for any of the treatments, in any of the intestinal regions (Table 6).

**Table 6:** Dry matter (DM) content, trypsin activity and bile salt levels in digesta of Atlantic salmon

	HFM	LFM	LFM +	LFM +	LFM +	LFM +	LFM +	HPS	HPS +	HPS +	
			Tauro-cholate	Bovine bile salt	Taurine	Lecithin	Suppl Mix		Bovine bile salt	Suppl Mix	Pooled SEM
*Digesta dry matter (mg/g)*											
PI1	10.1	9.3	8.4	8.2	9.0	10.7	9.9	9.1	9.2	10.4	0.54
PI2	11.9	11.0	10.3	9.6	11.5	12.1	11.5	12.3	10.5	12.1	0.58
MI	14.1	12.9	12.2	11.2	12.6	13.8	13.1	13.2	12.8	14.0	0.54
DI1	14.0^a^	12.9^ab^	11.4^bcd^	11.2^cd^	12.3^bcd^	13.1^ab^	11.8^bcd^	10.9^c^	12.8^abc^	11.6^bcd^	0.38
DI2	11.8^ab^	11.0^bc^	9.3^de^	9.7^cde^	10.7^bcd^	12.1^ab^	10.0^cd^	8.3^e^	9.4^cde^	8.1^e^	0.32
*Digesta trypsin activity (U/mg DM)*											
PI1	308^abc^	232^bc^	221^c^	293^abc^	234^bc^	226^c^	234^bc^	335^a^	330^ab^	344^a^	29
PI2	182^ab^	113^bc^	132^bc^	175^abc^	108^bc^	93^c^	158^bc^	155^bc^	192^ab^	249^a^	24
MI	106	72	72	139	78	53	87	78	126	120	30
DI1	50^abc^	38^c^	47^abc^	79^a^	41^bc^	37^c^	67^abc^	56^abc^	74^ab^	64^abc^	10
DI2	7^c^	8^c^	25^b^	48^a^	6^c^	10^c^	37^ab^	35^ab^	37^ab^	41^a^	5
*Digesta bile salt levels (mg/g DM)*											
PI1	290	355	376	367	356	285	268	330	346	299	75
PI2	228	262	256	303	268	292	290	242	301	251	57
MI	171	218	205	207	275	220	275	159	183	204	44
DI1	78	74	98	103	124	63	83	97	99	92	34
DI2	5	10	11	11	16	5	15	15	14	17	7

Different letters denote diet groups that are significantly different

## Discussion

### Effects of basal diet

In the present study, the two plant based diets (LFM and HPS) suppressed fish growth performance as compared to the high fish meal control diet (HFM). Animal growth parameters are arguably among the most appropriate and practical response variables for examining the effects of variation in diet composition, and a large body of literature has described effects of inclusion of fish meal alternatives on fish growth performance (reviewed by [32, 33]). The lower growth rates observed for the LFM and HPS diets were probably related to, or directly caused by the observed histomorphological changes in the distal intestine and the accompanying signs of gut dysfunction. Slight to moderate signs of enteritis in the distal intestine were observed in 2 and 10 out of 10 fish in the LFM and HPS fed fish, respectively. The changes were typical of soybean meal induced enteritis (SBMIE), including decreased enterocyte vacuolization, apical displacement of enterocyte nuclei, leukocyte infiltration of the epithelia and submucosa, and hyperplastic connective tissue in the lamina propria and submucosa [30, 31]. The histomorphological changes in the soya (HPS) fed fish were milder than what is typically observed in feeding trials with Atlantic salmon fed full-fat or extracted soya. The reason for this is not clear, but a likely explanation could be that the soy variant used in the current study contained lower levels of antinutrients, such as saponins.

The observed histomorphological alterations in fish fed the LFM and HPS diets were accompanied by alterations in a panel of indicators of gut function, most pronounced for the HPS groups. Decreased faecal dry matter, first reported in salmon by van den Ingh and co-workers [30], is frequently observed during distal intestinal inflammations and is probably a result of impaired ability to absorb water in the inflamed and damaged intestine. Decreased weight of the DI, as seen for fish fed the HPS diet, is also typically observed in concert with intestinal inflammation, apparently due to loss of intestinal mucosa [34]. Brush border membrane enzyme activity is a sensitive indicator of enterocyte dysfunction and changes may be present even in the absence of altered tissue histology. The lower LAP activity in fish fed HPS diets is in agreement with presence of SBMIE [3]. The higher trypsin activity, as seen for fish fed the HPS diet, is also commonly observed during intestinal inflammatory conditions. Trypsin activity in DI digesta typically shows a correlation with the severity of morphological changes associated with SBMIE [3, 35, 36]. Furthermore, the two plant diets tended to reduce blood plasma cholesterol and bile acid levels. This is a commonly observed response to plant feed ingredients, with their content of fibers, phytosterols, phytoestrogens and saponins that all may affect cholesterol and bile salt absorption from the intestine [37]. Increased levels of plant ingredients in formulated feeds will also reduce the dietary load of cholesterol and bile acids, as demonstrated in the present study. Dietary supplementation of plant based fish feeds with cholesterol and/or bile components have therefore been subject to systematic investigations, in order to assess if removal of marine based feed ingredients may create a deficiency of any of these compounds. These results are discussed in the following section.

### Effects of supplements

The main finding of the present work was that supplementation with bile salts, either as pure taurocholate or as a mix of bovine bile salts did not reduce signs of enteritis in the distal intestine. Similarly, supplementation with lecithin, taurine or combinations of bile acids, cholesterol and lecithin were also ineffective in ameliorating enteritic changes. The results of the present investigation contrast previous reports in rainbow trout [5, 18, 19], where supplementation with bovine bile salts (1.5–2.0 %), taurocholate (1.0 %) or soybean lecithin (2.0 %) were reported to prevent SBM-induced morphological abnormalities in the DI. Bile salt supplementation was also interpreted to restore growth, feed efficiency and intestinal maltase activity to comparable levels reported in the control group [5]. In contrast, the present study clearly indicated that supplementation with bovine bile salt at similar levels (1.8 %), either alone or in the suppl mix, impaired intestinal function. The strongest responses were seen when the bovine bile salt was supplemented to the LFM diet, as witnessed by reduced DI relative weight and LAP activity, increased trypsin activity in DI digesta and clear signs of enteritis in the DI. A likely explanation for the observed negative effects would be cytotoxic actions of the bile salts on intestinal mucosa. Toxicity of bile acids is thought to be highly correlated with its hydrophobicity. Therefore, the high levels (45 %) of unconjugated, more hydrophobic bile acids in the bovine bile salt may be the reason for the observed negative responses. The bovine bile salt also reduced fish growth. Feed intake was not monitored during the trial, and therefore, supplementation effects on palatability cannot be assessed. However, it is possible that the bovine bile salt may have caused reduced palatability.

There are likely direct interactions between bile acids and intestinal immunity. For example, in mammalian IBD models, activation of the nuclear receptor farnesoid X receptor (FXR) by natural or synthetic ligands has been associated with immunosuppressive actions and preservation of intestinal epithelial barrier integrity [38]. Specifically, FXR activation has been shown to decrease epithelial permeability and suppress proinflammatory cytokine levels in murine intestinal mucosa [17]. Additionally, FXR activation is inhibited by proinflammatory stimuli in different model systems [16]. As bile acids are natural endogenous FXR ligands, this may point to a direct role of bile salts in modulation of immune responses via FXR. Whether a similar mechanism exists in fish is currently unknown. However, a recent study demonstrated that FXR mRNA levels are strongly induced in the DI of Atlantic salmon suffering from SBMIE, concurrent with a marked induction of proinflammatory cytokines and alterations in bile salt metabolism [2, 39].

Reported studies of dietary inclusion of bile salts to teleost fish feeds are, to our knowledge, limited to the above mentioned studies with rainbow trout and our present work with salmon. In contrast, many studies have investigated dietary supplementation with cholesterol, taurine and lecithin/phospholipids to formulated fish feeds, in order to estimate requirements (reviewed in [20, 40, 41]). Reported results are not consistent, but positive effects of inclusion on fish growth and feed intake have often been observed, most prominent during larval and early juvenile stages. For adult fish, reported studies are fewer, but requirements of dietary cholesterol, taurine and lecithin/phospholipids are probably species-specific and absorption along the gut dependent on diet formulation. Importantly, evaluation of gut health and function, for example by histological examination, has not been carried out in most of these studies. Given that future fish feeds most likely will contain lower levels of cholesterol, bile salts and marine phospholipids, more research within this area is clearly warranted.

## Conclusions

In conclusion, dietary supplementation with bile components or lecithin in general did not improve endpoints regarding performance or gut health in Atlantic salmon, in clear contrast to what has been previously reported for rainbow trout. Thus, our work does not add to the understanding of a relationship between intestinal inflammation and deficiencies of bile acids and taurine. The reason why our results differ from previous observations in rainbow trout may be the general bile salt status of the fish and/or species differences. Follow-up studies are needed to clarify if lower levels of bile salts and cholesterol may give different and beneficial effects, and if other supplements and other combinations of supplements might improve the inflammation status of the distal intestine. The present study complements our previous report from the same feeding trial [42] and demonstrates how dietary cholesterol and bile salts are taken up and metabolized in Atlantic salmon, showing clear effects of the dietary supplements on gall bladder bile acid levels, cholesterol lipoprotein profiles and plasma levels of oxysterols and markers for cholesterol synthesis and breakdown.
